# Hydrocephalus-associated trigeminal neuralgia

**DOI:** 10.3389/fneur.2026.1687097

**Published:** 2026-01-26

**Authors:** Jiangwei Ding, Yangyang Wang, Xiaoyan Hao, Xinxiao Li, Hongliang Jiao

**Affiliations:** 1 Department of Neurosurgery, First Affiliated Hospital of Zhengzhou University, Zhengzhou, Henan, China; 2 Department of Pain Medicine, First Affiliated Hospital of Zhengzhou University, Zhengzhou, Henan, China; 3 Department of Neurology, First Affiliated Hospital of Zhengzhou University, Zhengzhou, Henan, China; 4 Department of Neurosurgery, Fifth Affiliated Hospital of Zhengzhou University, Zhengzhou, Henan, China

**Keywords:** Chiari malformation, hydrocephalus, pain, trigeminal neuralgia, ventriculoperitoneal shunt

## Abstract

**Background:**

Trigeminal neuralgia (TGN) secondary to hydrocephalus is relatively uncommon in clinical practice. This study aimed to investigate the correlation between hydrocephalus and TGN and evaluate the efficacy of surgical intervention in alleviating TGN.

**Methods:**

We conducted a retrospective analysis of three cases from our institution and performed a systematic literature review (PubMed search up to December 2024). The inclusion criteria were patients with concurrent hydrocephalus and TGN. Data were analyzed for demographic characteristics, treatment modalities, and outcomes.

**Results:**

Among the 21 analyzed cases (including the 3 patients reported in our study), the mean age was 38 years (range: 22–64), with a balanced gender distribution (male-to-female ratio: 11:10). The etiologies included isolated hydrocephalus (*n* = 12 cases), Chiari I malformation (CIM) (*n* = 5), Dandy–Walker syndrome (DWS) (*n* = 2), and tumor-related cases (*n* = 2). Ventriculoperitoneal shunt (VPS) resulted in complete pain relief in 75% (*n* = 9/12) of hydrocephalus cases, while endoscopic third ventriculostomy (ETV) was effective in two cases. Microvascular decompression (MVD) showed variable efficacy, with better outcomes when combined with cerebrospinal fluid (CSF) diversion procedures.

**Conclusion:**

Hydrocephalus may represent an underrecognized secondary cause of TGN. CSF diversion procedures (VPS/ETV) should be considered as first-line interventions, with MVD reserved for refractory cases. These findings support a multidisciplinary approach to diagnosis and management.

## Introduction

Trigeminal neuralgia (TGN) is a prevalent neurological disorder. Vascular compression is widely acknowledged as the predominant etiological factor in the development of TGN (1–3), while cerebellopontine angle lesions, such as epidermoid cysts, meningiomas, and vestibular schwannomas, are secondary etiological factors (4–7). However, a limited number of cases of hydrocephalus associated with TGN have also been reported (8–10). The primary clinical manifestation of hydrocephalus is increased intracranial pressure. When a patient meets both the imaging criteria for hydrocephalus and the established clinical criteria for TGN, their condition is designated as hydrocephalus-associated TGN. Hydrocephalus-associated TGN remains rare, with only sporadic reports since its first description in 1977 (8–10). Accordingly, we conducted a comprehensive analysis of 2,985 patients with TGN who were admitted to our medical center between October 2019 and December 2024, from which three cases of hydrocephalus were identified (approximately 1 in 1000).

## Methods

First, we described the clinical data of three patients with TGN complicated by hydrocephalus, as detailed in the Case Description section. Using PubMed, we then conducted a systematic literature search of studies published up to 12 December 2024. The search terms “trigeminal neuralgia” and “hydrocephalus” or “Chiari I malformation” were selected from the thesaurus of the National Library of Medicine. The search included articles in English or Chinese published between 1 November 1956 and 3 October 2024, without including subheadings or tags. The clinical data of the three cases were approved by the patients who were followed up through outpatient visits or telephone calls. Records were screened by Jiangwei Ding and evaluated by Yangyang Wang and Xiaoyan Hao with respect to the inclusion and exclusion criteria. Discrepancies were discussed and resolved by Hongliang Jiao or all reviewers.

### Inclusion and exclusion criteria

The objective of this study was to investigate the potential association between hydrocephalus and TGN. Consequently, patients diagnosed with both conditions were deemed eligible for inclusion in the study. We excluded articles not written in English or Chinese and non-original research studies, such as reviews and meta-analyses. All articles were critically reviewed and evaluated by experts from the Departments of Neurosurgery, Pain, and Neurology.

## Results

**Figure 1:**
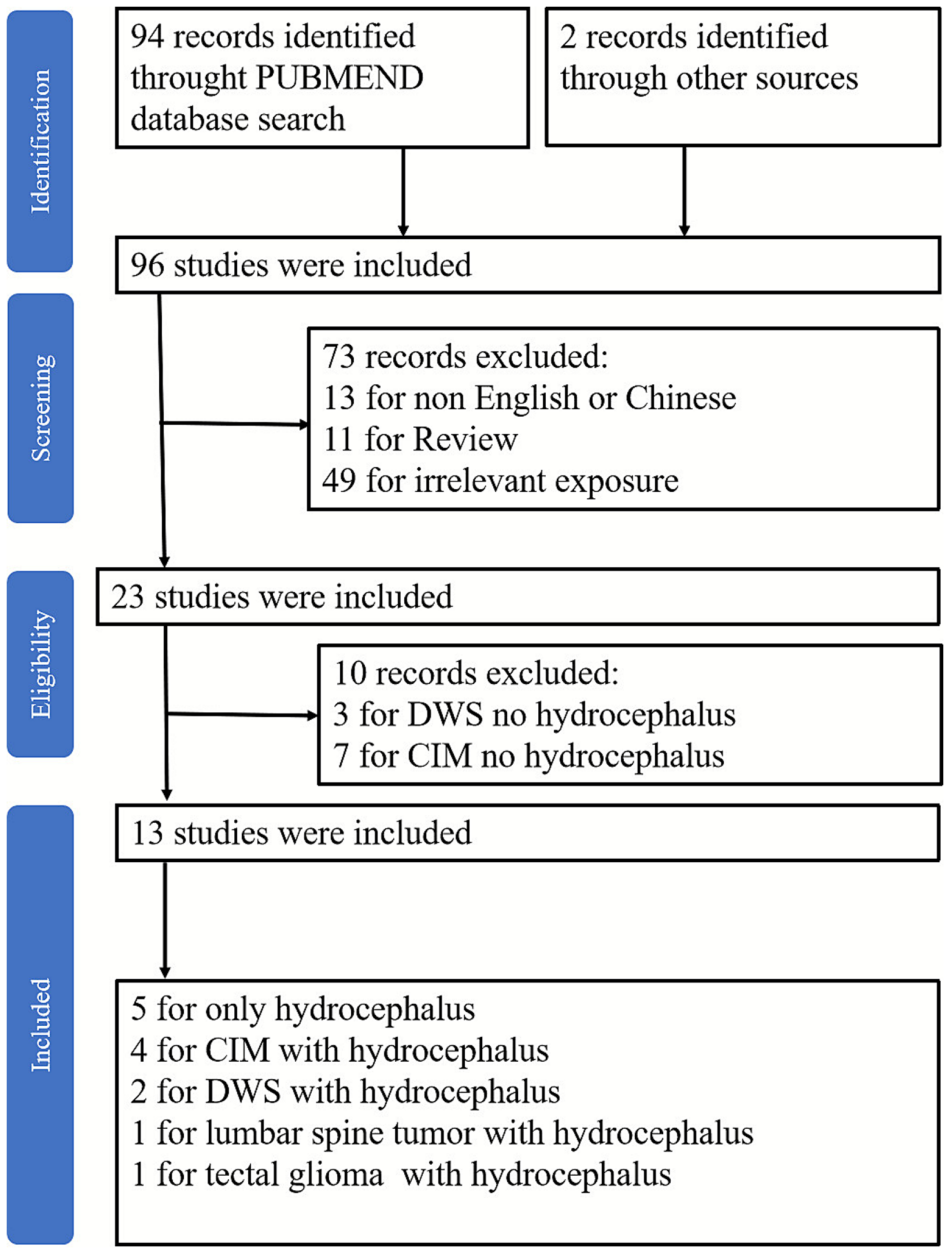
Flow diagram depicting the search process and study selection. DWS, Dandy–Walker syndrome; CIM, Chiari I malformation.

The literature search yielded 96 articles. After screening all abstracts, 83 records were excluded from the analysis. Finally, 13 studies met the inclusion criteria (Figure 1)—namely, studies that involved patients with hydrocephalus and TGN. Of the included studies, five were on hydrocephalus alone, four were on Chiari I malformation (CIM) with hydrocephalus, and two on Dandy–Walker syndrome (DWS) with hydrocephalus. The other two articles focused on hydrocephalus secondary to a lumbar tumor and a tectal glioma. A total of 21 patients, including the 3 cases reported in this study, were included in the analysis (Tables 1, 2). Of the 21 cases reviewed, more occurred in men than in women (male-to-female ratio: 11:10). The patients’ mean age was 38 years (range: 22–64 years; median: 36 years). In 9 patients, early oral administration of carbamazepine (CBZ) provided partial pain relief. Overall, 12 patients had hydrocephalus combined with TGN, 5 had CIM, 2 had DWS, 1 had a lumbar tumor, and 1 had a tectal glioma. Among the 12 patients with simple hydrocephalus, 8 underwent shunt placement and 2 underwent endoscopic third ventriculostomy (ETV). Two patients first underwent microvascular decompression (MVD), followed by abdominal ventriculoperitoneal shunt (VPS). Pain improvement was achieved in all hydrocephalus cases (complete relief: 75%; medication-controlled: 25%). Of the five patients with CIM, two underwent VPS, one underwent ETV, and one underwent MVD and shunt. One patient underwent occipital compression, VPS, and MVD. The pain was completely relieved in all five patients. Doctors recommended VPS for two patients with DWS, but the patients refused surgical treatment and insisted on conservative drug management. The pain was completely relieved after tumor resection in one patient with a lumbar tumor. ETV in cases of a tectal glioma with hydrocephalus achieved hydrocephalus control with complete disappearance of TGN (Table 3).

**Table 1:** Clinical data from previously reported cases of hydrocephalus and trigeminal neuralgia.

Case	Study	Gender	Age (y)	Complaint	Drug therapy	Imaging and discovery	CSF pressure	Diagnosis	Surgical strategy	Follow-up
1	Maurice-Williams (1977) (10)	Female	47	Ache behind the right eye, with stabs of pain radiating over the right side of the forehead.	NA	Lumbar pneumoencephalography showed gross ventricular dilatation, but air failed to rise above the surfaces of the cerebral hemispheres.	175 mmH_2_O	Hydrocephalus and paroxysmal facial pain (TGN-like)	VPS	The patient presented with headaches and cognitive decline 7 years later. Concurrently, the right-sided facial pain recurred. Following the replacement of the valve system with a VPS, her symptoms improved significantly, and her facial pain resolved.
2	Maurice-Williams (1977) (10)	Female	22	Shooting pains in the left lower jaw (2 y).	At first, CBZ (800 mg/d) was effective; later, there was no effect.	Right carotid angiogram showed marked enlargement of the lateral ventricle.	NA	Hydrocephalus and paroxysmal facial pain (TGN-like)	ventriculoatrial shunt (VAS)	There was no recurrence of pain after 12 months.
3	Maurice-Williams (1977) (10)	Female	49	Cold sensation over the right cheek (10 mth).	NA	Carotid angiogram showed slight dilatation of the lateral ventricles.	NA	Hydrocephalus and facial numbness and paresthesia	VAS	Symptoms were completely relieved within 10 weeks.
4	Tucker et al. (1978) (11)	Female	29	Sharp jabbing pain in the jaw (3 y).	At first, CBZ was effective; later, there was no effect.	Carotid arteriogram showed symmetrical ventricular enlargement.	NA	TGN and obstructive hydrocephalus	VSP (first operation) and VPS (second operation)	There was no recurrence of pain for 4.5 years after surgery.
5	Tucker et al. (1978) (11)	Male	24	Left-sided TGN (10 y).	Dilantin and CBZ were effective; later, there was no effect.	Positive-contrast ventriculogram showed aqueduct stenosis.	NA	TGN and obstructive hydrocephalus	VPS	After shunt surgery, shunt dysfunction caused pain recurrence, and symptoms disappeared after shunt adjustment. There was no recurrence of pain during the 1.5-year follow-up.
6	Findler et al. (1982) (12)	Female	42	Chronic right-sided retro-orbital and facial pain (NA).	NA	Huge symmetrical dilatation of the lateral ventricles, with mild enlargement of the third ventricle.	NA	TGN and hydrocephalus	VPS	The patient’s pain was relieved after VPS. However, pain recurred 6 months later due to shunt dysfunction. This was relieved again after shunt adjustment.
7	Iseki et al. (1999) (13)	Female	58	Pain in the right upper jaw (11 y) and difficulty walking, disorientation, and markedly decreased memory.	At first, CBZ (200 mg/d) was effective; later, there was no effect.	CT and MRI examinations revealed a markedly enlarged third ventricle, consistent with hydrocephalus.	16.1 mmHg	TGN and normal pressure hydrocephalus	VPS	After the operation, the acute facial pain disappeared quickly, returning to the original level of trigeminal neuralgic pain. With 200 mg of daily CBZ intake, she has managed daily life without difficulty.
8	Gnanalingham et al. (2005) (14)	Male	31	Right-sided facial pain that affected the lower jaw and gum (6 m).	At first, CBZ (600 mg/d) was effective; later, CBZ (1,200 mg/d) had no effect.	MRI brain scans revealed marked enlargement of the lateral and third ventricles, consistent with obstructive hydrocephalus and herniation of the cerebellar tonsils.	NA	TGN, obstructive hydrocephalus, and CMI	VPS	At 4–5 weeks postoperatively, TGN dramatically resolved, and the patient was slowly weaned off medications.
9	Teo et al. (2005) (15)	Female	38	Progressively worsening right-sided facial pain (3 y).	CBZ followed by gabapentin resulted in some improvement.	Preoperative sagittal T1-weighted MRI, showing hydrocephalus and Chiari I malformation.	NA	TGN, hydrocephalus, and CMI	ETV	After 3 months, symptoms had not recurred.
10	Schwartz et al. (2005) (16)	Female	36	Severe lancinating right-sided tongue and face pain (10 m).	NA	MRI demonstrated enlargement of the lateral and fourth ventricles. A diagnosis of spinal block was made, and a full spine MRI was obtained. An enhancing sausage-shaped mass was observed within the canal from the L1 to the L4 vertebral bodies.	NA	TGN, hydrocephalus, and spinal cord tumor	L1–L4 laminectomy and debulking of an extensive intradural tumor	Postoperative brain MRI showed a moderate decrease in ventricle size. The patient’s tongue and facial pain greatly improved.
11	Vince et al. (2010) (17)	Female	50	Weakness and paresthesia of both arms, gait ataxia, suboccipital headaches, retro-orbital pain, and left-sided triggerable neuralgic pain (NA).	NA	CT and MRI showed hydrocephalus and Chiari type I malformation.	NA	TGN, hydrocephalus, and CMI	Craniocervical decompression, VPS, and MVD	There was no recurrence of pain 3 years after MVD.
12	Zhang et al. (2013) (18)	Male	36	Paroxysmal lancinating pain confined to the left zygoma and cheek (2 m).	Tegretol (CBZ) 600 mg/d was used.	MRI revealed the bilateral lateral ventricles and the fourth and third ventricles were significantly enlarged with severe obstructive hydrocephalus, a huge posterior fossa cyst connected with the fourth ventricle, and hypoplastic vermis.	NA	TGN, hydrocephalus, and DWS	VPS was recommended, but the patient refused surgery.	NA
13	Liu et al. (2014) (9)	Male	24	Left-sided facial pain affecting the lower jaw and upper gum (5 y); involuntary paroxysmal contractions of ipsilateral face (6 m).	NA	Brain MRI revealed enlargement of the lateral and third ventricles. It also demonstrated Chiari I malformation, with the cerebellar tonsils herniating to the level of C1 and flattening of the skull base (platybasia).	NA	TGN, HS, hydrocephalus, and CMI	VPS	After the procedure, the patient’s HS was immediately resolved, and TGN disappeared 4 days after VPS. There were no postoperative complications. During the 4-month follow-up, there was no recurrence of TN or HFS.
14	Na et al. (2017) (8)	Male	31	Severe intermittent right-sided facial pain (3 m).	CBZ, pregabalin, and gabapentin were used without adequate symptom control.	MRI revealed hydrocephalus secondary to obstruction of the aqueduct of Sylvius, with marked dilation of the third and lateral ventricles.	NA	TGN and hydrocephalus	ETV	Six months postoperatively, the medication was progressively discontinued, and the pain symptoms fully resolved.
15	Na et al. (2017) (8)	Male	45	Left-sided TGN (NA).	CBZ (1,200 mg/d) was used without adequate symptom control.	MRI revealed incidental finding of triventriculomegaly, consistent with significant arrested supratentorial hydrocephalus with a web in aqueduct.	NA	TGN and hydrocephalus	ETV	The pain was alleviated immediately after surgery but subsequently recurred, localized to the corners of the mouth and the cheek area. Pain was controlled with medication.
16	Na et al. (2017) (8)	Female	50	Right-sided TGN (NA).	NA	MRI revealed a communicating hydrocephalus.	NA	TGN and communicating hydrocephalus	MVD; VPS	Two years later, the patient developed recurrent attacks of disequilibrium, nausea, and vomiting, as well as recurrence of neuralgic (electric-shock-like pain) symptoms. The TGN disappeared after VPS.
17	Musa et al. (2021) (19)	Female	23	Sudden, severe left orofacial pain, mainly localized around the mouth (NA).	CBZ and diclofenac were effective.	MRI demonstrated a hypoplastic cerebellar vermis that was displaced and rotated superiorly; an enlarged cystic posterior fossa communicating with the fourth ventricle; and the presence of hydrocephalus.	NA	TGN, hydrocephalus, and DWS	Shunt was recommended, but the patient refused surgery.	NA
18	Gabay et al. (2024) (20)	Female	28	Left-sided TGN (18 m).	CBZ was ineffective.	Tectal glioma and obstructive hydrocephalus.	NA	TGN, hydrocephalus, and tectal glioma	ETV	The pain did not recur after 4 months.

TGN, trigeminal neuralgia; CBZ, carbamazepine; MRI, magnetic resonance image; HS, hemifacial spasm; Y, years; mth: months; CSF, cerebrospinal fluid; VPS, ventriculoperitoneal shunt; VAS, ventriculoatrial shunt; VCS, ventriculo-cisternal shunt; NA, not available; ETV, endoscopic third ventriculostomy; MVD, microvascular decompression; DWS, Dandy–Walker syndrome; CIM, Chiari I malformation.

**Table 2:** Clinical data from our cases of hydrocephalus and trigeminal neuralgia.

Case	Gender	Age (y)	Complaint	Drug therapy	Imaging and discovery	CSF pressure	Diagnosis	Surgical strategy	Follow-up
1	Male	31	Paroxysmal pinprick pain in the right cheek (1 y)	At first, CBZ (100 mg/d) was effective; later, CBZ (400 mg/d) had no effect.	Brain MRI revealed enlargement of the supratentorial ventricular system, with the posterior margin of the cerebral aqueduct either adhered to or obstructed. The fourth ventricle appeared normal in size, while the cerebellar tonsils were herniated below the level of the foramen magnum.	240 mmH_2_O	TGN and hydrocephalus	VPS	No recurrence of TGN was observed during the 5-year follow-up period.
2	Male	64	Right trigeminal neuralgia (1 y)	Drugs were less effective.	Brain MRI revealed enlargement of the bilateral lateral ventricles and the third ventricles with interstitial edema as well as normal structure of the fourth ventricle.	130 mmH_2_O	TGN and hydrocephalus	MVD and VPS	The patient’s TGN was relieved after the operation, and no TGN recurred after 3 years and 10 months of follow-up.
3	Male	35	Pain affecting the left upper and lower lip, the cheek, and the lower teeth (6 mth).	CBZ proved ineffective.	Brain MRI revealed dilatation of the bilateral ventricles, accompanied by hydrocephalus and interstitial edema. There was also evidence of cerebellar tonsillar herniation and an empty sella.	340 mmH_2_O	TGN, hydrocephalus, and CMI	MVD and VPS	TGN was significantly relieved 1 year after MVD, and facial numbness and TGN disappeared 1 month after the second surgery (VPS).

TGN, trigeminal neuralgia; CBZ, carbamazepine; MRI, magnetic resonance image; Y, years; CSF, cerebrospinal fluid; VPS, ventriculoperitoneal shunt; ETV, endoscopic third ventriculostomy; MVD, microvascular decompression; DWS, Dandy–Walker syndrome; CIM, Chiari I malformation.

**Table 3:** Etiological distribution and treatment outcomes.

Etiology	Cases (*n*)	Treatment outcomes
Isolated hydrocephalus	12	Complete relief: 75%; medication controlled: 25% (VPS: 8; ETV: 2; MVD + VPS: 2).
CIM	5	All complete relief (VPS/ETV/MVD)
DWS	2	Conservative management
Tumor-related	2	Complete relief after resection

ETV, endoscopic third ventriculostomy; MVD, microvascular decompression; DWS, Dandy–Walker syndrome; CIM, Chiari I malformation; VPS, ventriculoperitoneal shunt.

### Case description

#### Case 1

We present the case of a 30-year-old man who presented with a 1-year history of paroxysmal pinprick-like pain in the right cheek. During episodes—each lasting approximately 30 min—the patient experienced difficulty opening his mouth, brushing his teeth, and eating. Initially, the symptoms were significantly alleviated by oral administration of CBZ (100 mg/day). However, at the current visit, the patient reported that the pain relief was no longer adequate, despite an increase in the dose to 400 mg/day. Initially, the patient was treated in the Department of Stomatology under the assumption that the condition was related to impacted wisdom teeth. However, extraction of the impacted wisdom teeth did not alleviate pain. Brain MRI revealed enlargement of the supratentorial ventricular system, with the posterior margin of the cerebral aqueduct appearing either adherent or obstructed (Supplementary materials). The fourth ventricle appeared normal in size (Supplementary materials). The lumbar cerebrospinal fluid (CSF) pressure was 240 mmH_2_O. After discussions with patients and their families, it is recommended that VPS be initially performed. Should this prove ineffective, trigeminal MVD should be considered as a subsequent procedure. Postoperative computed tomography (CT) revealed a reduction in ventricular size compared with before surgery (Supplementary materials). The patient experienced pain relief after surgery, without pain recurrence, during the 5-year follow-up period.

#### Case 2

We present the case of a 64-year-old man who presented with a 5-year history of right TGN and dizziness that had persisted for the previous 2 months. Two months before this presentation, he underwent trigeminal nerve MVD at an external medical facility (December 2020). After surgery, the right facial pain and dizziness showed slight improvements after local infiltration and administration of analgesic medications. However, the patient subsequently experienced worsening dizziness along with symptoms of unsteady gait and disorientation. Past history: The patient initially underwent trigeminal nerve MVD in 2019. Despite the procedure, significant postoperative pain persisted. Hydrocephalus was identified at that time; however, given the absence of symptoms of hydrocephalus, a VPS was not indicated. In 2016, the patient experienced a large cerebellar infarction, which was managed conservatively. After admission, brain MRI revealed enlargement of the bilateral lateral and third ventricles with interstitial edema and a normal structure of the fourth ventricle (Supplementary materials). The lumbar CSF pressure was 330 mmH_2_O. Thus, VPS was performed (March 2021). Postoperative CT revealed that the ventricles were smaller than before the operation (Supplementary materials). The patient’s TGN was relieved after the operation, and no TGN recurrence was observed after 46 months of follow-up.

#### Case 3

**Figure 2:**
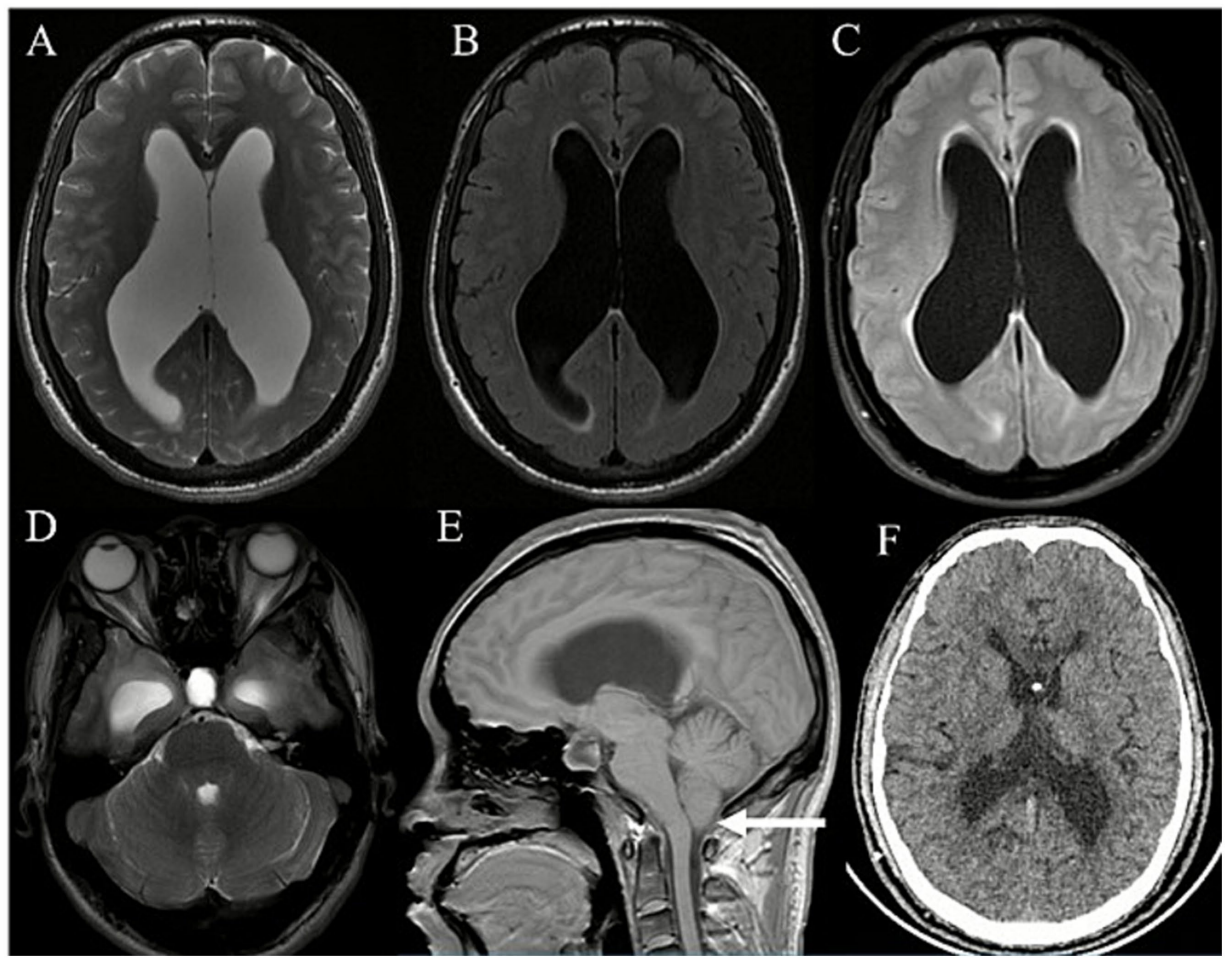
Prior to MVD, brain MRI revealed bilateral ventricular enlargement **(A,B)** with mild interstitial brain edema **(B)**. Post-MVD and pre-VPS brain MRI revealed persistent bilateral ventricular enlargement, exacerbated interstitial brain edema **(C)**, and a normal-sized fourth ventricle **(D)**. In addition, there was evidence of cerebellar tonsillar herniation [**(E)**; white arrow]. Post-VPS brain CT indicated a reduction in ventricular size **(F)**.

We present the case of a 35-year-old man who presented with a 6-month history of pain that affected the left upper and lower lips, cheek, and lower teeth, which had become aggravated for 1 month. The pain was described as sharp and cutting, similar to that caused by a knife wound. Pain attacks typically lasted for several seconds to minutes and were characterized by sudden onset and cessation. An intermittent period of pain relief occurred between attacks, during which facial stimulation did not trigger any pain. Symptoms were exacerbated by wind exposure. Oral administration of CBZ proved ineffective. The clinical diagnosis was TGN. Brain MRI revealed dilatation of the bilateral ventricles accompanied by hydrocephalus and interstitial edema (Figures 2A,B). Evidence of cerebellar tonsillar herniation and an empty sella was also found. As the patient exhibited no signs of hydrocephalus, only trigeminal MVD was performed. After the operation, the patient achieved complete remission of the TGN. One year after surgery, the patient experienced episodic visual disturbances characterized by blurred, darkened, and diplopic vision that lasted approximately 10–20 s. These symptoms were partially alleviated by eye rubbing or blinking. Concurrently, the patient reported facial numbness on the right side, which preceded the visual disturbances and lasted approximately 10–20 s. Reevaluation of the brain MRI scan revealed bilateral ventricular dilation, exacerbated periventricular edema, and tonsillar herniation (Figures 2C–E). Lumbar puncture revealed a CSF pressure of 340 mmH_2_O. After the slow release of approximately 30 mL of CSF, the visual disturbances were significantly alleviated. A VPS procedure was successfully performed. Postoperative CT imaging revealed a significant reduction in ventricular size compared with before surgery (Figure 2F). After the operation, the patient’s symptoms of blurred vision were relieved immediately, but the numbness of the right facial area persisted until 1 month after the operation.

## Discussion

TGN and hydrocephalus are prevalent neurological disorders (2, 21). However, the concurrent occurrence of TGN in patients with hydrocephalus, or hydrocephalus-associated TGN, is clinically uncommon (8–10). Since 1977, when Maurice Williams first reported two cases of TGN with hydrocephalus (10), approximately 21 such cases—including our three cases—have been documented in the literature (Tables 1, 2). Whether this coexistence is coincidental or causally related remains a subject of significant concern and ongoing investigation. Our investigations have suggested a potential association, as shunt (VPS, ventriculoatrial shunt, and ventriculo-cisternal shunt) or ETV alone alleviated TGN in the majority of patients, while shunt obstruction led to symptom recurrence, which was subsequently resolved after shunt adjustment (10–12). Of the 21 cases, 12 had simple hydrocephalus combined with TGN. Among these 12 patients, 8 underwent shunt and 2 underwent ETV. Two patients underwent MVD first and VPS later. The pain improvement rate was 100% in 12 patients, of whom it was completely relieved in 9 patients (75%). Among the 20 patients, 1 had a lumbar tumor that led to secondary hydrocephalus and TGN. After tumor resection, postoperative brain MRI revealed reduced ventricular size, and the symptoms of TGN were significantly alleviated (16).

In addition, we identified five cases combined with CIM (9, 14, 15, 17) and two cases combined with DWS (18, 19). Whether hydrocephalus led to cerebellar tonsillar herniation or if the latter caused hydrocephalus remains unclear. Fortunately, VPS was successfully performed in the five patients with hydrocephalus, effectively alleviating their neuralgia. Symptoms of TGN were also observed in patients with CIM without hydrocephalus. These patients underwent suboccipital decompression for severe spinal cord compression or secondary syringomyelia, which resulted in the significant relief of TGN postoperatively (22–25). DWS may present with or without hydrocephalus (19, 26). The literature lacks evidence of the effectiveness of hydrocephalus shunting for alleviating TGN secondary to DWS. Zhang and Musa et al. (18, 19) reported a case of DWS complicated by secondary hydrocephalus that led to TGN. However, the patient declined surgical intervention. Gabale et al. (27) described a patient who developed pain only after undergoing VPS, with symptoms eventually relieved after vascular decompression. In contrast, radiofrequency thermocoagulation rhizotomy was effective in managing TGN in patients with DWS without hydrocephalus, providing substantial pain relief (26).

Each of the three patients we have reported thus far presented a unique course of treatment and recovery. One patient underwent a VPS, which resulted in a complete improvement of the symptoms. The other two patients initially underwent MVD because of their lack of understanding of the disease. One of these patients did not experience complete symptom relief after two MVD procedures. After this, the patient underwent a VPS, which ultimately led to a complete improvement of the symptoms. The third patient also began with MVD. While the surgery successfully alleviated the pain symptoms, the patient later developed facial numbness and blurred vision. The onset of these symptoms followed a chronological pattern, with paroxysmal facial numbness occurring first, followed by blurred vision. Eventually, after undergoing VPS, all symptoms were completely relieved.

While vascular compression is the established primary cause of classic TGN and MVD is its cornerstone treatment (28), the mechanism of hydrocephalus-associated TGN is multifactorial and likely involves a combination of the following pathways:

1 Brainstem and trigeminal nerve traction from ventricular enlargement

Hydrocephalus causes caudal displacement of the brainstem and distortion of the perimesencephalic cisterns. This displacement can exert mechanical traction on the trigeminal nerve root entry zone or along its cisternal segment. The efficacy of CSF diversion in relieving TGN strongly supports this mechanism, as shunt placement reverses the displacing forces. This traction hypothesis is analogous to the proposed mechanism of TGN following extensive anterior temporal lobectomy, whereby brain shift places traction on the trigeminal nerve (29). Vince et al. (17) documented a case of bilateral TGN attributed to brainstem descent stretching the trigeminal nerves.

2 Secondary vascular displacement and neurovascular compression

Ventricular enlargement and brainstem displacement can alter the anatomical relationship between neighboring vessels and the trigeminal nerve. A vessel not originally compressing the nerve may be displaced into a compressing position. Conversely, existing neurovascular conflicts might be exacerbated. This explains why some patients, like the one in our series, experience partial or temporary relief from MVD. However, if the primary pathology (hydrocephalus) persists, displacement forces may reconfigure the vascular anatomy, leading to recurrence or incomplete response, as noted in Cases 2 and 3, where definitive relief came only after CSF diversion.

3 Trigeminal nucleus dysfunction from raised intracranial pressure and brainstem distortion

Elevated intracranial pressure and direct deformation of the brainstem may affect the trigeminal sensory nucleus complex within the pons. Irritation or compression of the nucleus itself can generate paroxysmal pain signals. Khaleghi et al. (30) examined TGN relief via MVD targeting vascular compression on the trigeminal sensory nucleus and proved this structure’s role in pain generation. Furthermore, conditions such as CIM with syringobulbia or high cervical stenosis causing upper cord/brainstem distortion can present with TGN, often relieved by posterior fossa or cervical decompression rather than MVD (22–25, 31). This indicates that intrinsic brainstem pathology, which is potentially induced by hydrocephalic distortion, can be a sufficient cause.

**Figure 3:**
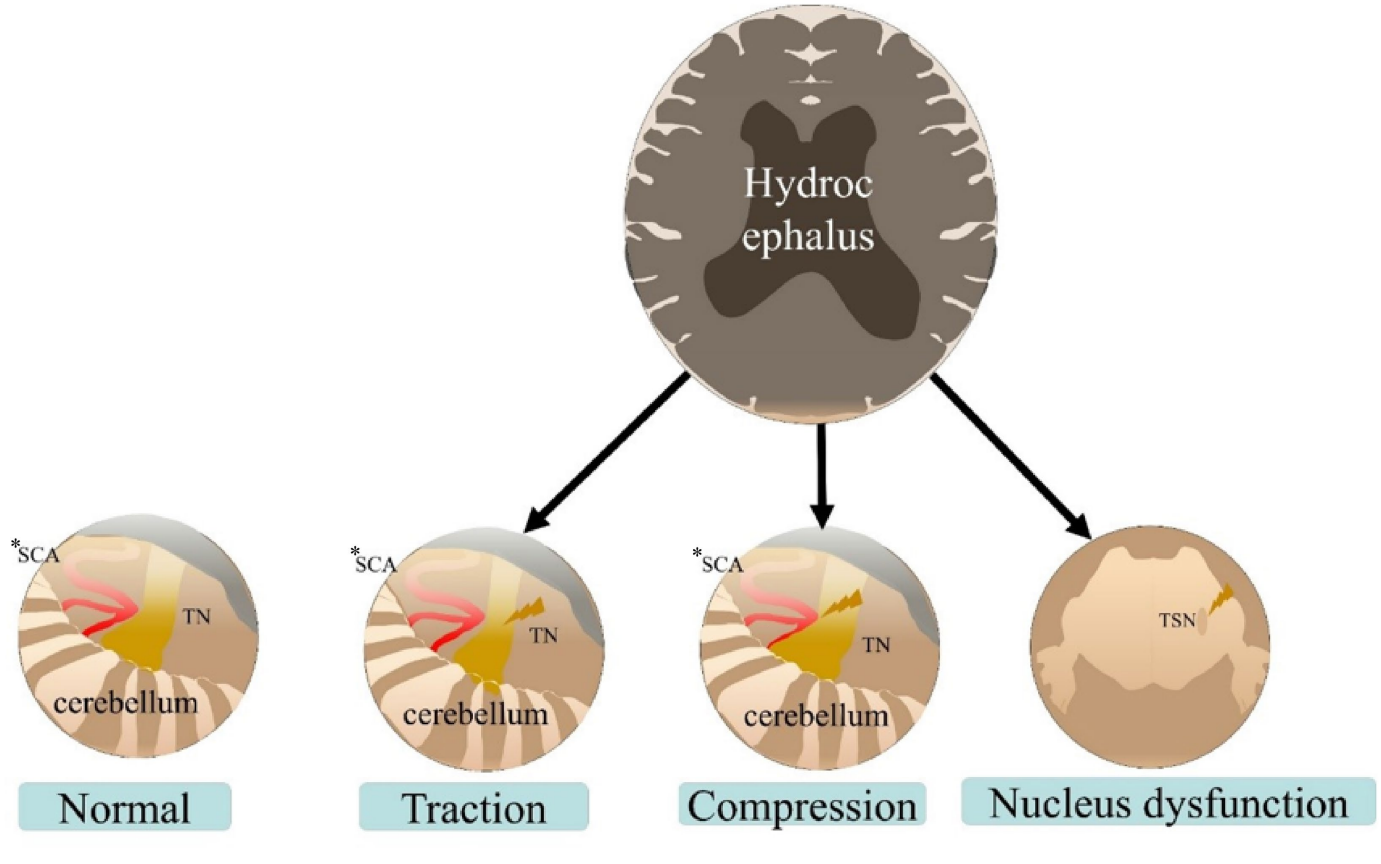
Potential mechanism of hydrocephalus-associated trigeminal neuralgia. SCA, superior cerebellar artery; TN, trigeminal nerve; TSN, trigeminal sensory nucleus. *The responsible arteries include, but are not limited to, the SCA.

The mechanisms—traction, secondary vascular compression, and nuclear irritation—are not mutually exclusive and likely coexist in varying degrees in hydrocephalus-associated TGN (Figure 3). The dominant mechanism may vary per patient, influencing the treatment response.

**Figure 4:**
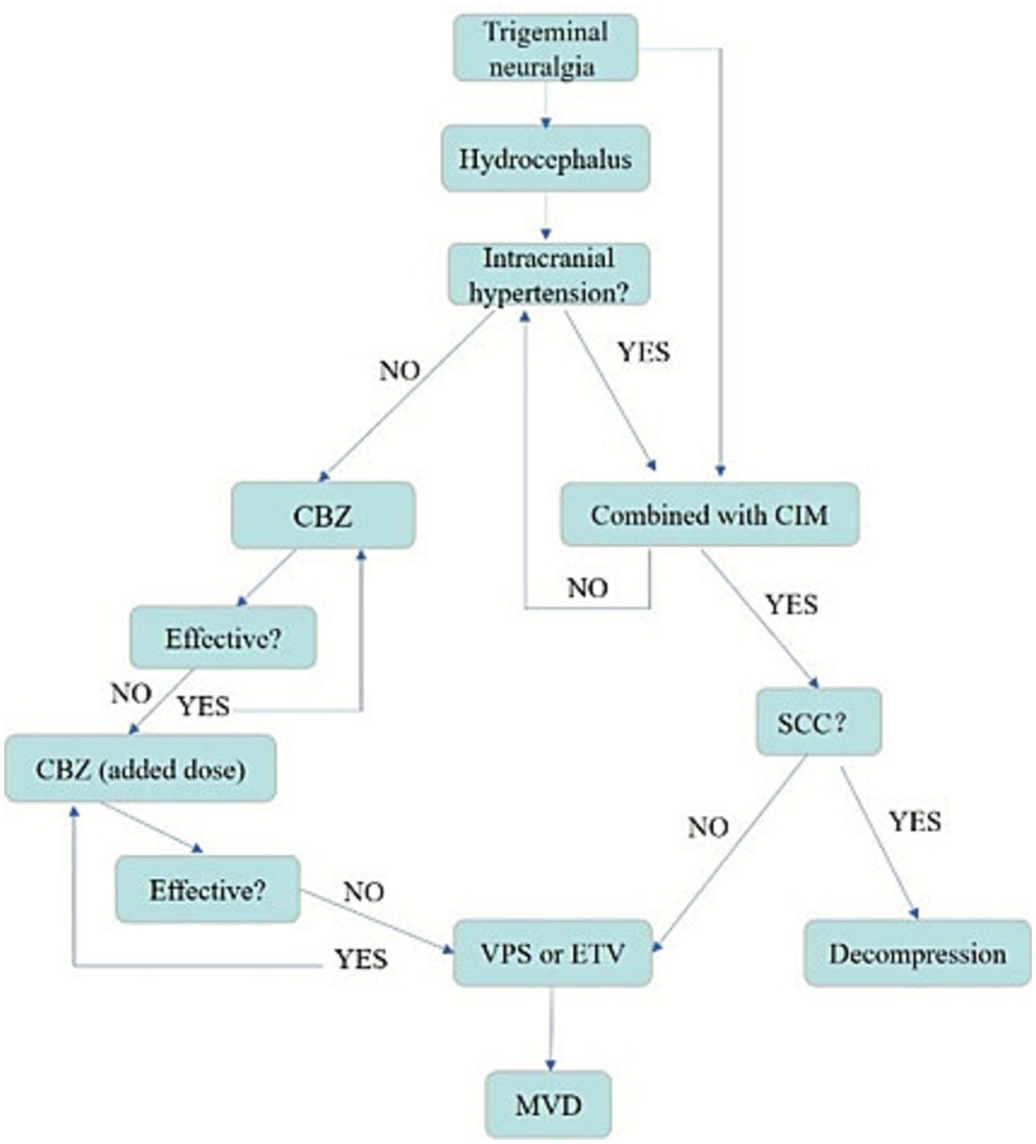
Therapeutic strategy framework for hydrocephalus-associated trigeminal neuralgia. CBZ, carbamazepine; CIM, Chiari I malformation; SCC, spinal cord compression; VPS, ventriculoperitoneal shunt; ETV, endoscopic third ventriculostomy; MVD, microvascular decompression.

Therefore, a paradigm shift in management is recommended. While CBZ remains a first-line symptomatic treatment, the primary intervention for hydrocephalus-associated TGN should address CSF dynamics. For patients with clear hydrocephalus, especially with symptoms of intracranial hypertension, such as headache, nausea, visual disturbance, and gait instability, CSF diversion (VPS or ETV) should be the initial surgical consideration. MVD should be reserved for cases in which TGN persists after effective CSF diversion or a clear, dominant neurovascular conflict is identified independently of hydrocephalus. In cases of CIM with significant cord compression or syringomyelia, suboccipital decompression is the priority. Figure 4 summarizes this proposed therapeutic strategy.

## Limitations

The sample size of 21 patients, which included our three cases, was limited. Due to the small sample size and the significant heterogeneity in the reviewed cases, this study provided limited high-level evidence-based medical data. In addition, there was a lack of standardized measurements of the severity of pain and hydrocephalus. Due to an insufficient understanding of both conditions, the coexistence of hydrocephalus and TGN may have been underrecognized and significantly underestimated. Thus, it is plausible that the prevalence of hydrocephalus concurrent with TGN is higher than what is currently appreciated, necessitating further comprehensive evaluation through multicenter, cross-regional, and international collaborations. Future research could also explore randomized controlled trials.

## Conclusion

While the co-occurrence of hydrocephalus and TGN is clinically uncommon, an association exists between the two conditions that poses unique challenges for healthcare providers. This combination of conditions requires a thorough and systematic approach to diagnosis and treatment. In clinical settings, when patients present with hydrocephalus, clinicians should meticulously inquire about any facial paresthesia, particularly the presence of TGN. For patients diagnosed with TGN, brain MRI should be conducted as needed to rule out the possibility of coexisting hydrocephalus, thereby preventing delayed diagnosis and disease progression. In cases where hydrocephalus and TGN coexist, it is crucial to evaluate the potential correlation between the two conditions. Hydrocephalus may be a potential etiological factor of TGN. Multidisciplinary consultations involving neurologists, neurosurgeons, and pain specialists should be sought if necessary. For patients diagnosed with hydrocephalus-associated TGN, following a thorough multidisciplinary assessment, initial consideration should be given to procedures such as VPS or ETV. If these measures prove ineffective, MVD may be considered a subsequent treatment option.

In summary, hydrocephalus represents an important secondary cause of TGN that requires distinct management approaches. CSF diversion procedures should be prioritized, with MVD considered for refractory cases. This paradigm shift in treatment strategy may improve outcomes for this challenging patient population.

## Data Availability

The original contributions presented in the study are included in the article/Supplementary material; further inquiries can be directed to the corresponding author.
