# Supplementary material for: Hydrocephalus-associated trigeminal neuralgia
Source: Front Neurol. 2026 Jan 26;17:1687097. doi: 10.3389/fneur.2026.1687097 (PMC12883382; doi:10.3389/fneur.2026.1687097)
Supplement: Supplementary file 1 [file Data_Sheet_1.zip › Supplementary Materials/Figure Case1 and Case2.doc]

**Figure 1-Case1.**Preoperative magnetic resonance imaging (MRI) scans (blackwater imaging) revealed significant enlargement of the bilateral ventricles and interstitial brain edema(A), and dilation of the third ventricle(B), while the fourth ventricle remained normal in size(C). Postoperative CT demonstrated a reduction in the size of the bilateral ventricles(D).

**Figure 2-Case2.** Preoperative magnetic resonance imaging (MRI) scans (blackwater imaging) revealed significant enlargement of the bilateral ventricles and interstitial brain edema(A), and enlargement of the third ventricle(B), while the fourth ventricle remained normal in size(C). Postoperative CT demonstrated a reduction in the size of the bilateral ventricles(D).
